# Examining the Association and Directionality between Mental Health Disorders and Substance Use among Adolescents and Young Adults in the U.S. and Canada—A Systematic Review and Meta-Analysis

**DOI:** 10.3390/jcm7120543

**Published:** 2018-12-13

**Authors:** Sarvenaz Esmaeelzadeh, John Moraros, Lilian Thorpe, Yelena Bird

**Affiliations:** 1 School of Public Health, University of Saskatchewan, Saskatoon, SK S7N 2Z4, Canada; sarv.esmaeelzadeh@usask.ca (S.E.); john.moraros@usask.ca (J.M.); 2 FRCP, Faculty, Community Health and Epidemiology, University of Saskatchewan, Saskatoon, SK S7N 2Z4, Canada; lilian.thorpe@usask.ca

**Keywords:** depression, anxiety, alcohol, cannabis, tobacco, adolescents, young adults, U.S., Canada

## Abstract

Background: The purpose of this systematic review was to examine the association and directionality between mental health disorders and substance use among adolescents and young adults in the U.S. and Canada. Methods: The following databases were used: Medline, PubMed, Embase, PsycINFO, and Cochrane Library. Meta-analysis used odds ratios as the pooled measure of effect. Results: A total of 3656 studies were screened and 36 were selected. Pooled results showed a positive association between depression and use of alcohol (odds ratio (OR) = 1.50, 95% confidence interval (CI): 1.24–1.83), cannabis (OR = 1.29, 95% CI: 1.10–1.51), and tobacco (OR = 1.65, 95% CI: 1.43–1.92). Significant associations were also found between anxiety and use of alcohol (OR = 1.54, 95% CI: 1.19–2.00), cannabis (OR = 1.36, 95% CI: 1.02–1.81), and tobacco (OR = 2.21, 95% CI: 1.54–3.17). A bidirectional relationship was observed with tobacco use at baseline leading to depression at follow-up (OR = 1.87, CI = 1.23–2.85) and depression at baseline leading to tobacco use at follow-up (OR = 1.22, CI = 1.09–1.37). A unidirectional relationship was also observed with cannabis use leading to depression (OR = 1.33, CI = 1.19–1.49). Conclusion: This study offers insights into the association and directionality between mental health disorders and substance use among adolescents and young adults. Our findings can help guide key stakeholders in making recommendations for interventions, policy and programming.

## 1. Background

Mental health disorders and substance use are important problems and relatively common among adolescents and young adults [1]. These two conditions are the leading cause of years lived with disability worldwide [2]. People are more likely to initiate substance use during adolescence, a critical period in one’s life as they transition from childhood to adulthood [3]. It is estimated that 50% of all mental health disorders start by the age of 14 years old and 75% by the age of 25 years old [4]. Mental health disorders and substance use problems are closely linked, and both conditions can share similar biological and environmental components [5,6]. 

Mental health disorders contribute significantly to the burden of disease [2]. Globally, approximately one in five adolescents experience a mental health disorder each year [7]. Depressive and anxiety disorders are quite prevalent among adolescents and young adults. In the U.S., 8.4% of adolescents were diagnosed with either anxiety or depression in their lifetime [8]. In Canada, 5% of adolescents were diagnosed with an anxiety disorder and 6.3% with a mood disorder, mainly depression [9]. Despite the magnitude of the problem posed by mental health disorders among adolescents and young adults in the U.S. and Canada, frequently they are undiagnosed and when diagnosed not treated properly [10]. 

Adolescents and young adults with mental health disorders are at an increased risk of many negative health and social outcomes including self-harm and suicide [11], poor academic performance [12], and involvement in risky behaviors and poor sexual health [13]. It is reported that there is a link between adverse life events such as maltreatment and violence, loss, intra-familial problems, and school and interpersonal problems and suicidal behavior among young people. Also, the number of negative life events experienced by a young individual is reported to have a positive dose–response relation to suicidal behavior [14]. Risk factors for mental health disorders include biological factors (such as genetic tendency, head trauma, and substance abuse), psychological factors (such as maladaptive personality traits, difficult temperament, and physical/sexual abuse), and social factors (such as family conflict, loss of a loved one, bullying, and academic failure) [15].

Substance use among adolescents and young adults is a growing public health concern in developed countries such as the U.S. and Canada. Alcohol, cannabis, and tobacco are the substances most commonly used by adolescents and young adults. In Canada, the highest prevalence of alcohol (83%), cannabis (30%), and tobacco (18%) use was seen among young adults aged 20 to 24 years old followed by adolescents aged 15 to 19 years old (alcohol: 59%, cannabis: 21%, and tobacco: 10%) [16]. Similar trends in the prevalence of substance use were seen in the U.S. [17]. The magnitude of substance use among adolescents and young adults represents a major public health concern. 

Substance use among adolescents and young adults is associated with many negative health, social, and economic consequences. These vary from suicidal behaviors [18] to low academic performance and school dropout [19], employment problems later in life [20], and risky behaviors [21]. Moreover, initiation of substance use in adolescence may be associated with the development of substance abuse and substance dependence later in life [22]. Risk factors associated with substance use are found at the individual (such as age, sex, personality and family history of substance use), interpersonal (such as relationship with parents, siblings, and peer pressure), and environmental (such as social norms, availability and accessibility of substance) levels [23].

Evidence suggests a complex interplay between substance use and vulnerability to mental health disorders [23,24]. Mental health disorders and substance use can co-occur due to a variety of reasons, including but not limited to: (1) common risk factors (such as environmental, genetic, personality, biological factors or traumatic events); (2) self-medication hypothesis (coping with psychological pressures and stressors as they relate to maturation, untreated traumas, and underlying conditions may lead to substance use and related risky behaviours); (3) substance use-induced mental health problems (individuals may develop a mental health problem as a direct (i.e., pharmacogenic) consequence of their substance use); and (4) substance use-related mental health problems (individuals may develop a mental health problem as an indirect (i.e., socio-economic stressors, unemployment, dysfunctional relationships) consequence of their substance use) [23,24]. 

Previous studies have been conducted examining the relationship between mental health disorders and substance use. Some studies have suggested that an association exists between depression and/or anxiety and alcohol, cannabis or tobacco use [25,26,27,28,29,30]. However, findings in the literature have been inconsistent [31,32,33,34,35,36]. Due to the high prevalence of mental health disorders and substance use among adolescents and young adults and the high potential of co-occurrence, further research in this important area is warranted. The purpose of this study was to examine the association and directionality between the three most common substances used (alcohol, cannabis, and tobacco) and two mental health disorders (depression and anxiety) among adolescents and young adults in the U.S. and Canada. Specifically, its objectives aimed to determine and quantify the association and directionality between: (1) depression and alcohol use, (2) depression and cannabis use, (3) depression and tobacco use, (4) anxiety and alcohol use, (5) anxiety and cannabis use, and (6) anxiety and tobacco use among adolescents and/or young adults.

## 2. Methods

### 2.1. Data Sources

We conducted an extensive systematic literature review on the following electronic databases: Medline, PubMed, Cochrane Library, Embase, and PsycINFO. Our searches consisted of two major categories (substance use behaviors and mental health disorders) and their corresponding Medical Subject Headings (MeSH) terms and keywords: Subject OR Title (alcohol drinking or alcohol use or cannabis or marijuana smoking or marijuana use or smoking or tobacco smoking or tobacco use) AND Subject OR Title (depression or depressive disorders or major depressive disorder or anxiety or anxiety disorders or mood disorders). Snowball method (reference tracking) was also used to scan the reference lists of retrieved full-text articles and then to search manually for additional relevant literature. 

### 2.2. Eligibility Criteria, Data Extraction and Analysis

The inclusion criteria for this review centered on the following: (1) English language peer-reviewed articles, available in full text, with human studies, published from 2000 to 2017. All study designs were eligible except for case series and case report. Newspaper, conference posters, dissertations were excluded; (2) depression/anxiety symptoms or disorders (major depressive disorder, panic disorder, social anxiety disorder, specific phobias, generalized anxiety disorder) data measured by using standardized scales, diagnostic criteria, self-reported surveys or diagnosed by healthcare professionals; (3) substance use (alcohol, cannabis, or tobacco) or disorder data presented, analyzed, and discussed; (4) target population that included adolescents and/or young adults; (5) only studies conducted in the US or Canada; and (6) data was either presented as an odds ratio (OR) or permitted the OR to be calculated.

Two screeners, independently, reviewed the list of identified articles to assess eligibility for inclusion in our study. Any disagreements were resolved by a tiebreaker vote. Extraction criteria included: year of publication, study design, sample size, target population, country where the study was conducted, measurement instrument used for substance use and mental health disorders, effect measure, covariates, and results. The effect measures examined the association between any of the following categories: (1) depression and alcohol use; (2) depression and cannabis use; (3) depression and tobacco use; (4) anxiety and alcohol use; (5) anxiety and cannabis use, and (6) anxiety and tobacco use. If a study discussed more than one of these categories, we extracted and analyzed all relevant effect measures separately. 

### 2.3. Definitions of Mental Health Disorders and Substance Use

Overall, the definitions and measurements of mental health disorders and substance use in the various studies included in our review were heterogeneous. When multiple measures were used, we selected the most standardized and commonly used measure in order to maximize comparability between studies (Appendix A) [25,26,27,28,29,30,31,32,33,34,35,36,37,38,39,40,41,42,43,44,45,46,47,48,49,50,51,52,53,54,55,56,57,58,59,60].

Standardized scales for depression or anxiety symptoms, instruments measuring depression or anxiety disorders using diagnostic criteria, and diagnosis of depression or anxiety by healthcare professionals were used by the various studies to define and select for mental health disorders. Certain studies examined subgroups of anxiety disorders such as social phobia or panic attack. We counted these subgroups as anxiety disorders and included them in our analysis. Similarly, a variety of well-established definitions and measurements were employed by a number of studies for substance use. For example, some studies defined participants as current users, whereas others as regular, daily, or ever users in their measurement categories (tobacco, alcohol and/or cannabis use). Appendix A provides the details for the definitions of mental health disorders and substance use employed by the various studies that were included in our analysis.

### 2.4. Quality Assessment

The modified Newcastle-Ottawa Scale (NOS) was used to assess the quality of both cross-sectional and longitudinal study designs. This scale includes three components and 8 items: (1) selection of study groups (4 items); (2) comparability of the groups (1 item); and the (3) ascertainment of the outcomes of interest (3 items) [61]. To assess the quality of the studies a star system was used. The quality of each study was determined by assigning it to one of three subgroups: (1) good (≥2 stars for selection of study groups, 1 star for comparability of the groups, and 3 stars for ascertainment of the outcomes of interest components); (2) fair (1 star for selection of study groups and 2 stars for ascertainment of the outcomes of interest components); or (3) poor (0 stars for selection of study groups, 0 stars for comparability of the groups, and ≤1 star for ascertainment of the outcomes of interest components). Risk of bias was designated as: low, if there was good quality in all components; unclear/moderate, if there was fair quality in one or more components without poor quality in any components; or high, if there was poor quality in any one of the components. 

### 2.5. Meta-Analysis

Statistical analysis was completed using the Comprehensive Meta-Analysis software version 3 (Biostat Inc., Englewood, NJ, USA). For pooled mean effect size, random-effects models were used because differences were expected in the methodology and the samples of selected studies [62]. The primary outcome measure was odds ratio (OR). ORs and 95% confidence intervals (CI) were either extracted from the articles or calculated by the authors using the quantitative data provided in the studies. Six separate meta-analyses were conducted. Q-statistics and *I*^2^ were used to examine the heterogeneity among studies. Publication bias was assessed by graphing funnel plots to visualize any possible asymmetries and Duval and Tweedie’s trim and fill test was conducted to adjust the results in case of publication bias [63]. Sensitivity analysis was done using one-study removed analysis. Moreover, subgroup analysis was performed using moderator variables including point estimate (crude or adjusted), target population (adolescents and young adults), severity of mental health disorders (symptoms and disorders), and intensity of substance use behavior (definitions).

## 3. Results

### 3.1. Study Selection and Characteristics

In total, 3656 articles (Medline (*n* = 945), PubMed (*n* = 883), Cochrane Library (*n* = 41), Embase (*n* = 1618), and PsycINFO (*n* = 169)) were identified by our initial search. After removing studies prior to the year 2000 and duplicates, 2341 articles remained. Title and abstract screening excluded 2177 articles, leaving 164 for full-text screening. After the full-text screening, 30 articles remained. Six additional articles were manually added by use of the snowball method. 

In completing the title, abstract, and full-text screening, studies were excluded, if they were related to: (1) exposures other than those of interest in this study, such as cessation/withdrawal from substances, use of e-cigarettes, opiates, cocaine, methamphetamine, sedative, and hallucinogens; (2) outcomes other than those of interest in this study, such as suicide ideations, bipolar disorder, mania, postpartum depression, and hypomania; (3) the recruited population was other than that of interest in this study, such as adults, participants who were pregnant, specific ethnic groups or gender, military veterans, participants with comorbid chronic medical illnesses such as diabetes, cardiovascular or lung diseases. 

**Figure 1:**
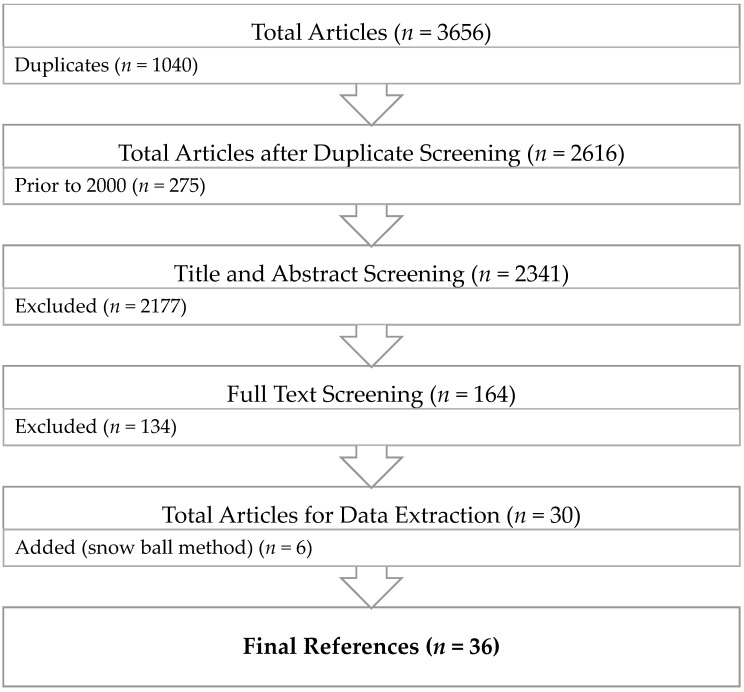
PRISMA diagram.

All 36 selected articles used either a cross-sectional (*n* = 19) or longitudinal (*n* = 17) study design. The summary of our study selection is shown in a PRISMA diagram (Figure 1). Study characteristics are presented in Table 1. The quality of studies was assessed using a modified Newcastle-Ottawa scale (NOS), which found 5 studies with a low, 27 with a moderate, and 4 with a high risk of bias.

### 3.2. Synthesis of Results

The results of the meta-analysis found significant positive associations between depression and alcohol use (OR = 1.50, 95% CI: 1.24–1.83), cannabis use (OR = 1.29, 95% CI: 1.10–1.51) and tobacco use (OR = 1.65, 95% CI: 1.43–1.92). Similarly, significant positive associations were found between anxiety and alcohol use (OR = 1.54, 95% CI: 1.19–2.00), cannabis use (OR = 1.36, 95% CI: 1.02–1.81) and tobacco use (OR = 2.21, 95% CI: 1.54–3.17) (Table 2). 

### 3.3. Analysis of the Directionality

**Figure 2:**
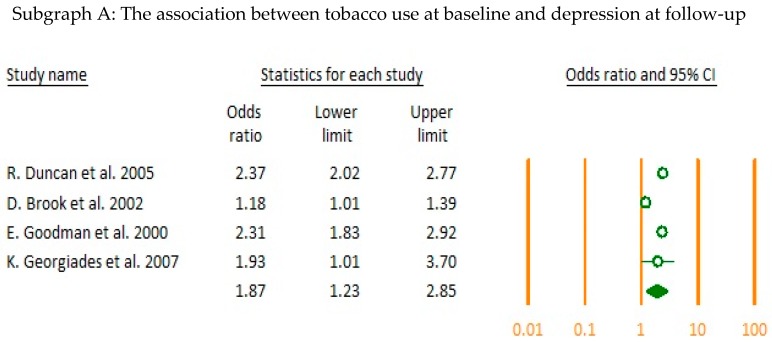

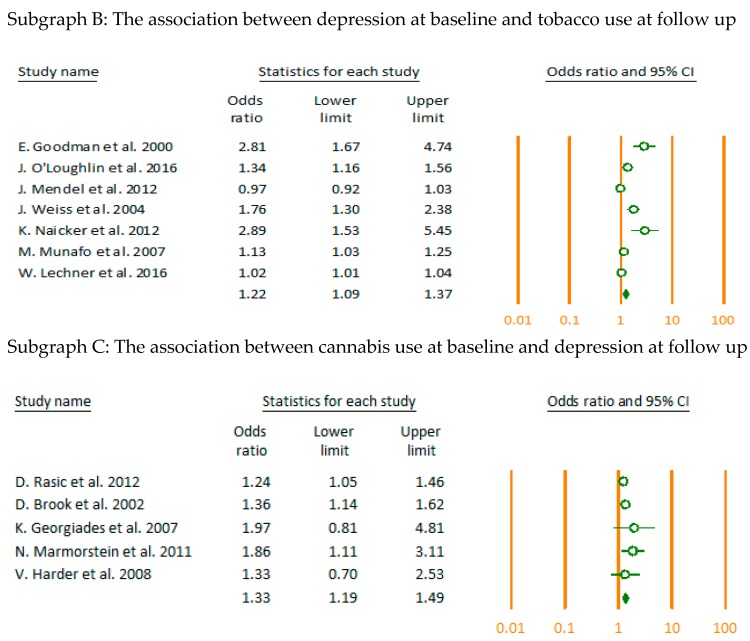
Examining the directionality in longitudinal studies (forest plots A–C).

The directionality of the association between each relevant category was assessed by pooled analysis of the longitudinal studies. Results showed that there was a significant positive bi-directional association between tobacco use at baseline leading to depression at follow-up (OR = 1.87, CI = 1.23–2.85) and depression at baseline leading to tobacco use at follow-up (OR = 1.22, CI = 1.09–1.37). Cannabis use at baseline was significantly associated with depression at follow-up (OR = 1.33, CI = 1.19–1.49) (Figure 2). 

### 3.4. Subgroup Analysis

Due to the heterogeneity detected upon pooled analysis, subgroup analysis was conducted to examine whether the differences within categories could be attributed to specific moderators (age group, measures of mental health disorder, and severity of substance use). Results of our subgroup analysis are summarized in Table 3. Results from the subgroup analysis by age group including adolescents (aged 10 to 18 years old) and young adults (aged 18 to 24 years old) found that the association between mental health disorders and substance use remained significant among adolescents but not among young adults. Subgroup analysis based on severity of mental health disorders showed a consistently higher point estimate for clinically diagnosed mental health disorder patients. Finally, subgroup analysis based on the intensity of substance use showed a recurring theme, whereby, as severity increased, mental health disorders also increased (Table 3).

### 3.5. Publication Bias

Funnel plots showed no evidence of publication bias in the included studies that examined the association between depression and alcohol use; depression and cannabis use; depression and tobacco use; anxiety and alcohol use; and anxiety and tobacco use. However, there was evidence of publication bias in the included studies which examined the association between anxiety and cannabis use. Therefore, in these instances, a Duval and Tweedie’s trim and fill method was used to test and adjust for the publication bias in our meta-analysis. 

## 4. Discussion

Our findings suggest that a significant association exists between depression and the use of alcohol, cannabis, and tobacco. Similarly, significant associations were found to exist between anxiety and the use of alcohol, cannabis, and tobacco. A bidirectional relationship was observed between depression and the use of tobacco. Additionally, we found evidence that cannabis use at baseline led to depression at follow-up. The results of our study were consistent with those reported in previous systematic reviews [64,65,66] and provide additional evidence in support of the inter-association between mental health disorders and substance use. 

Harmful alcohol use, which has been defined as heavy episodic or binge drinking, is an important health issue among adolescents and young adults in the U.S. [67]. The adolescent/young adult brain is still developing, and there is evidence to suggest that frequent use of alcohol at this age may lead to mental health disorders including depression [68,69]. Our results support these findings. We found that alcohol use was a significant predictor of higher levels of depression among adolescents and young adults. However, further research is needed in this area with more longitudinal studies, using longer follow-up periods. 

A systematic review conducted by Lev-Ran et al. found a positive association between cannabis use at baseline and the development of depression [70]. Our results corroborate these findings and demonstrate that the overall pooled estimate for depression among heavy cannabis users was higher, compared to light users. Another systematic review by Number-Kedzior et al. found a significant positive association between cannabis use at baseline and anxiety at follow-up [66]. In our study, it was not possible to infer whether such a causal relationship exists due to the lack of sufficient studies on this topic, which fit our inclusion criteria. Proposed mechanisms for the association between cannabis and depression could be due to biological effects from the alteration in neurotransmitters such as serotonin in the brain [71] and its impact on developing depression and mediating psychosocial factors such as unemployment and educational failure [50]. We observed that when adjusting for personal income and years of education, the association between cannabis use and depression was reduced [50].

In our study, we found a stronger pooled estimate for tobacco use predicting depression, compared to depression predicting tobacco use. Moreover, a stronger pooled estimate was observed in those studies that used a clinical measure of depression rather than depressive symptoms. Although, our study was limited in investigating the associations rather than the causality between mental health disorders and substance use, our results are biologically plausible. For instance, the association between tobacco use and anxiety or depression may be due to the effect of nicotine on the central neurotransmitter systems and peripheral circulating stress hormones [72]. In the brain, the primary target of nicotine (the addictive substance found in tobacco) is the neuronal nicotinic acetylcholine receptors (nACHRs). The nACHRs have a large number of subtypes responsible for the regulation of different neurotransmitter systems. Nicotine can make alterations in the balance of these neurotransmitter systems by activating or inactivating the receptors responsible for release of stimulatory (glutamate), inhibitory (GABA), and modulatory (dopamine, norepinephrine, and serotonin) neurotransmitters in different brain regions. Thus, different behavioral outputs such as depression or anxiety may occur [72]. The ability of nicotine to act as an anxiolytic or anxiogenic and depressant or anti-depressant agent is complex and may dependent on the severity and duration of its use, the route of administration, and the genetic and behavioral state of the individual user [72].

The effects of nicotine on the hypothalamic pituitary adrenal (HPA) axis are centrally mediated by the stimulation of neurotransmitter systems [73]. Acute nicotine exposure can result in increase in basal level of stress hormones by stimulating the HPA axis [73]. The anxiogenic effect of nicotine may be explained by the acute peripheral effect of tobacco smoking such as the increases observed in blood pressure, heart rate, and cortisol output. Also, nicotine increases corticosteroids in the amygdala, a brain area critical for emotionality. With chronic use of tobacco, nicotine alters activity of the HPA axis. The HPA axis in combination with genetic factors may be involved in producing tolerance to the effects of nicotine. Thus, this might predispose smokers to depressive episodes during withdrawal [72,74]. 

The association between anxiety and tobacco use might be explained by the negative effect of tobacco use on different brain pathways such as neurotransmitter systems, inflammation and the immune system, oxidative and nitrosative stress pathways, neurotrophins and neurogenesis regulations, mitochondrial function, and epigenetic influences. Alteration in abovementioned pathways can also play a role in development of anxiety disorders [75]. 

### 4.1. Strengths and Limitations

Our study has several strengths. There is a high level of congruence between our findings and those reported in the existing literature. However, this systematic review is unique because: (1) it incorporates only studies originating in the U.S. and Canada, (2) it focuses on the adolescent and young adult population, and (3) it examines the associations and directionality between depression or anxiety and alcohol, cannabis, or tobacco use, separately. 

Our study also has some limitations. We found that certain factors such as the study design (longitudinal vs. cross-sectional), the intensity of substance use (light vs. heavy), and the variation in clinical diagnosis (depressive and anxiety disorders vs. symptoms) affect the outcomes of our study. Participant’s age (adolescents vs. young adults) also influenced the inter-association between substance use behaviors and mental health disorders. In our review, several of our included studies used different instruments to measure depression and anxiety and a broad spectrum of measurements for substance use, making the results difficult to compare due to their heterogeneity. When extracting data, we were unable to calculate the unadjusted odds ratio from all included studies. Different studies varied in the degree to which they controlled for potential confounders. Variation in the population sampled, length of follow-up in longitudinal studies, and different sample sizes also added to the variability.

### 4.2. Implications for Practice, Research and Policy

The co-occurrence of mental health disorders and/or substance use presents complex challenges in their prevention, diagnosis, and treatment. If left untreated, these conditions may result in loss of productivity, poor educational outcomes, increased psychiatric hospitalizations/emergency department visits, inefficient use of limited healthcare resources, and a higher prevalence of chronic diseases. By affecting change at the personal (increase awareness), interpersonal (decrease stigma), and societal (policies and interventions) levels, we can assist adolescents and young adults to make better choices, seek support as needed, and live healthier and well-adjusted lives. 

Overall, our systematic review and meta-analysis found a clear association between depression/anxiety and substance use, regardless of the severity of the mental health disorder or the intensity of substance use. These findings have significant implications and may impact:

(1) Practice: our study helps highlight the importance of addressing adolescents and young adults’ mental health (depression/anxiety) and substance use behaviors (drinking, smoking, cannabis use) early, concurrently and with an integrated clinical approach through the use of timely screening, team-based diagnosis, patient-oriented treatment and effective interventions. These observations underscore the need for a shift in our collective perspective, when implementing integrated services for patients with co-occurring disorders.
(2) Research: our findings corroborate and expand those reported in previous studies and provide invaluable insight and guidance to future rigorous, longitudinal research that aims to widen our knowledge of psychopathology by elucidating the associated links and delineating best practices in the prevention, diagnosis, and treatment of co-occurring mental health disorders and substance use. Additionally, it will be of keen scientific interest to investigate and determine whether substance use cessation treatments lead to reduction and/or remission of depression/anxiety or whether the treatment of depression/anxiety leads to reduction and/or cessation of substance use among adolescents and young adults.
(3) Policy: our results demonstrate that integrated treatment approaches and health education campaigns are needed to improve quality of care and increase awareness among the public and healthcare practitioners of the associations between depression/anxiety and substance use among adolescents and young adults. School-based intervention programs, in particular, hold much promise as they could encourage adolescents and young adults to seek professional help safely and in a supportive environment.

### 4.3. Recommendations

Given the findings of our study, it is important that patients diagnosed with mental health disorder and/or a substance use problems be evaluated, screened, and if need be, treated for both conditions. To assist in this regard, the following recommendations are proposed:

(1) Bilateral cooperation between the U.S. and Canada: along with sharing the longest international boarder, the two countries share many common socio-cultural factors, demonstrate similar patterns in the prevalence of mental health disorders and substance use, and report common health priorities (i.e., advancement of mental health and substance use services) [76,77]. Therefore, it would be beneficial for key stakeholders at educational settings in both the U.S. and Canada to collaborate and exchange information and ideas on how to further improve their health education and promotion programs.
(2) Implementation of the quadrants of care model (the New York Model): in our systematic review, it became apparent that as the severity of mental health disorders and intensity of substance use varied, different approaches to their healthcare management were needed. Thus, the potential usefulness of the quadrants of care model to most appropriately direct the efforts of healthcare professionals [78]. Individuals treated simultaneously by two or more healthcare providers at one point of entry may receive an integrated treatment plan for both conditions. By following this model, individuals will receive an appropriate level of care based on their needs, and this will ensure the efficient use of available resources.
(3) Reforming the healthcare system: healthcare reforms are needed to make the necessary changes from the current parallel and independent practice sectors towards coordinated systems of care. Coordinated practice sectors will require sharing funds, developing mandates in concert, and treating affected individuals collaboratively. Reforms should be broad and encompass all levels of service delivery including health promotion and prevention, diagnosis, treatment, and research.
(4) Efficient use of limited resources: preventive interventions, early detection, diagnosis, and treatment of the co-occurrence of mental health disorders and substance use, can improve the quality of life of adolescents and young adults. Addressing this issue upstream will reduce the associated healthcare costs and permit the efficient use of limited resources, including the need for frequent psychiatric hospitalizations, over-use of emergency departments, and ambulatory care.
(5) Training of healthcare providers: it is essential to cross-train healthcare providers from different sectors to increase their awareness of the existing association between mental health disorders and substance use. There is a need for the characterization of patient risk profiles, the use of valid assessment tools, and updated diagnostic and treatment guidelines to help improve outcomes for these two conditions among adolescents and young adults.

## 5. Conclusions

This study offers significant insight into the association and directionality between substance use (alcohol, cannabis, and tobacco) and mental health disorders (depression and anxiety) among adolescents and young adults in the U.S. and Canada. Our findings can help guide key stakeholders (school administrators, nurses, physicians, public health professionals, and policymakers) in making evidence-based recommendations for school-based interventions, policy, and programming. It is increasingly evident that adolescents and young adults with substance use issues may also suffer from co-occurring mental health disorders. However, they are frequently undiagnosed and, consequently, they do not receive the specialized cross-treatment needed to address both conditions. To be most effective, school-based substance use and mental health programmes should consider integrating and expanding their services for patients with dual disorders. Further research is needed to help guide evidence-based treatment and initiatives for this vulnerable population.

## Figures and Tables

**Table 1:** Characteristics and associations of selected studies.

First Author’s Name, Year, Country	Sample Size	Target Population	Substance Use Measure	Mental Health Disorders Assessment	% of Depressive Participants	Controlled Variables	OR (95% CI)
**Association between Depression and Alcohol Use**							
**Cross-Sectional Studies**							
Elissa Weitzman, 2004, U.S. [55]	27,687	College students aged 18–24	Binge/non-drinkers	Depressive symptoms: The 5-item subscale of SF36	4.9%	Age, sex, ethnicity	Adjusted: 1.05 (0.96–1.14)
Martha Kubik, 2005, U.S. [33]	3466	Grade 7 students	Heavy/non-drinkers	Depressive symptoms: CES-D, cut-off point of 16.	35%	Age, ethnicity, socioeconomic status (SES), smoking, cannabis, and inhalants use	Adjusted: 2.03 (1.30–3.17)
Susan Roberts, 2009, U.S. [32]	418	College students	Binge/non-drinkers	Depressive symptoms: BDI-II, cut-off point of 20	22%	-	Crude: 0.99 (0.61–1.58)
Elisabeth Simantov, 2000, U.S. [48]	5513	Grade 7–12 students	Regular/non-drinkers	Depressive symptoms: Modified version of CDI, cut-off point of 9	18.2%	-	Crude: 2.08 (1.79–2.42)
Linda Richter, 2015, U.S. [26]	24,445	Adolescents aged 12–20	Binge/non-drinkers	Major depressive disorder: Diagnosed depression (self-reported)	-	Sex, ethnicity, and age	Adjusted: 1.45 (1.11–1.91)
Eleanor Hanna, 2001, U.S. [31]	2001	Adolescents aged 12–16	Moderate/non-drinkers	Major depressive disorder: Diagnostic interview schedule, DSM-III	9%	Age, sex, ethnicity, family poverty level, school problem, smoking status	Adjusted: 3.31 (1.39–7.90)
Longitudinal Studies: Alcohol Use (Exposure) → Depression (Outcome)							
Mallie Paschall, 2005, U.S. [44]	13,892	Grade 6–12 students	Moderate/non-drinkers	Depressive symptoms: CES-D, cut-off point of 16.	5.3%	-	Crude: 1.17 (1.11–1.23)
David Brook, 2002, U.S. [52]	736	Adolescents aged 14, 13-year follow-up	Ever/never alcohol users	Major depressive disorder: Modified version of CIDI	8.3%	Age, sex, parental educational level, family income, prior psychiatric disorders	Adjusted: 1.72 (1.35–2.20)
Longitudinal Study: Depression (Exposure) → Alcohol Use (Outcome)							
Kiyuri Naicker, 2012, Canada [51]	1027	Adolescents aged 16–17, 10-year follow-up	Heavy/non-drinkers	Major Depressive Disorder: CIDI-SF, cut-off point of 5	6.9%	Sex and SES	Adjusted: 1.78 (1.10–2.88)
**Association between Depression and Cannabis Use**							
Cross-Sectional Studies							
Lee Ridner, 2005, U.S. [40]	895	College students aged 18–24	Current/non- cannabis users	Depressive symptoms: CES-D, cut-off point of 16	-	-	Crude: 1.27 (1.00–1.61)
Susan Roberts, 2009, U.S. [32]	418	College students	Current/non- cannabis users	Depressive symptoms: BDI-II, cut-off point of 20	22%	-	Crude: 1.99 (1.23–3.22)
Martha Kubik, 2005, U.S. [33]	3466	Grade 7 students	Current/non- cannabis users	Depressive symptoms: CES-D, cut-off point of 16	35%	Age, ethnicity, SES, smoking, alcohol, and inhalants use.	Adjusted: 1.02 (0.66–1.57)
Longitudinal Studies: Cannabis Use (Exposure) → Depression (Outcome)							
Daniel Rasic, 2012, Canada [27]	976	Grade 10 students, 2-year follow-up	Current/non- cannabis users	Depressive symptoms: CES-D, cut-off point of 22 (M) and 24 (F)	20%	Sex	Adjusted: 1.24 (1.05–1.46)
Katholiki Georgiades, 2007, Canada [50]	1282	Adolescents aged 12–16, 14-year follow-up	Past 6-month/non- cannabis users	Major depressive disorder: CIDI-SF	11.8%	Physical health, life satisfaction, personal income, years of education	Adjusted: 1.97 (0.81–4.81)
David Brook, 2002, U.S. [52]	736	Adolescents aged 14, 10-year follow-up	Ever/never cannabis users	Major depressive disorder: Modified version of CIDI	8.3%	Age, sex, parental educational level, family income, prior psychiatric disorders	Adjusted: 1.36 (1.14–1.62)
Naomi Marmorstein, 2011, U.S. [56]	1252	Adolescents aged 17, A 6-year follow-up	CUD/non- cannabis users	Major depressive disorder: The structured clinical interview for DSM-III-R.	13.9%	MDD by age 17 and gender	Adjusted: 1.86 (1.11–3.11),
Valerie Harder, 2008, U.S. [53]	1494	Adolescents aged 12–16, 7-year follow-up	CUD/non- cannabis users	Major depressive disorder: CIDI, DSM IV diagnostic criteria	6%	Ethnicity, family income, free lunch, tobacco, alcohol, and other illegal drug use, parental monitoring, concentration, behavior problems, shyness, anxiety symptoms, intervention status	Adjusted: 1.33 (0.70–2.53)
**Longitudinal Study: Depression (Exposure)** → **Cannabis Use (Outcome)**							
Cynthia Suerken, 2014, U.S. [45]	3146	College students, A 6-month follow-up	Ever/never cannabis users	Depressive symptoms: CES-D, Iowa short form	-	Sex, ethnicity, parental education and spending money available, varsity athlete, club, intramural sports, member of a sorority/fraternity, attend religious services, live on campus, relationship status, current use of tobacco, alcohol, and lifetime use of other illicit drugs	Crude: 1.03 (1.01–1.05)
**Association between Depression and Tobacco Use**							
**Cross-Sectional Studies**							
Lee Ridner, 2005, U.S. [40]	895	College students aged 18–24	Current/non-tobacco users	Depressive symptoms: CES-D, cut-off point of 16	-	-	Crude: 1.49 (1.18–1.90)
Susan Roberts, 2009, U.S. [32]	418	College students, aged 18–21	Current/non- tobacco users	Depressive symptoms: BDI-II, cut-off point of 20	22%	-	Crude: 2.08 (1.29–3.36)
Elisabeth Simantov, 2000, U.S. [48]	5513	Grade 7–12 students	Regular/non- tobacco users	Depressive symptoms: Modified version of CDI, cut-off point of 9	18.2%	-	Crude: 2.47 (2.06–2.97)
Sung Chung, 2014, U.S. [25]	11,848	Grade 9–11 students	Current/non- tobacco users	Depressive symptoms: 1 item question	28.4%	-	Crude: 1.33 (1.16–1.53)
Shahm Martini, 2002, U.S. [46]	11,201	Adolescents aged 12–17	Current/non- tobacco users	Depressive symptoms: YSR, cut-off point of 3	-	Age, ethnicity, school attendance, site, substance use behaviors	Crude: 1.83 (1.67–2.01)
Carla Berg, 2008, U.S. [47]	299	Adolescents aged 10–19	Current/non- tobacco users	Depressive symptoms: A 5-item question, cut-off point of 21	12%	Age, sex, ethnicity, church attendance, perceived parental attitude	Crude: 3.83 (1.65–8.88)
Martha Kubik, 2005, U.S. [33]	3466	Grade 7 students	Current/non- tobacco users	Depressive symptoms: CES-D, cut-off point of 16	35%	Age, ethnicity, SES, all other substance use behaviors	Adjusted: 1.56 (1.14–2.14)
Gilat Grunau, 2009, Canada [28]	6943	Students aged 13–18	Current/non- tobacco users	Major depressive disorder: Prescribed depression medications	2.2%	Age, sex, ethnicity, parent(s), sibling(s), peer(s) smokes, anxiety	Adjusted: 2.59 (1.79–3.73)
Amanda Richardson, 2012, U.S. [49]	1884	Adolescents aged 12–15	Ever/never tobacco users	Major depressive disorder: NIMH-DISC-IV	-	Age, ethnicity, attending school, poverty index ratio, live with smokers, anxiety disorders	Adjusted: 2.80 (1.13–6.91)
Eleanor Hanna, 2001, U.S. [31]	719	Adolescents aged 12–16	Current/non- tobacco users	Major depressive disorder: Diagnostic interview schedule, DSM-III	9%	Age, sex, ethnicity, family poverty status, school problem, drinking status	Adjusted: 0.98 (0.33–2.90)
Longitudinal Studies: Tobacco Use (Exposure) → Depression (Outcome)							
Brian Duncan, 2005, U.S. [41]	13,068	Students grade 7–12, 1-year follow-up	Current/non- tobacco users	Depressive symptoms: CES-D, cut-off point of 22 (M) and 24 (F)	-	Disability, age, ethnicity, household, parental education, county-level variables	Crude: 2.37 (2.02–2.77)
Elizabeth Goodman, 2000, U.S. [42]	8704	Adolescents aged 11–22, 1-year follow-up	Current/non- tobacco users	Depressive symptoms: CES-D, cut-off point of 22 (M) and 24 (F)	6.4%	Age, sex, ethnicity, parental education	Crude: 2.31 (1.83–2.92)
Katholiki Georgiades, 2007, Canada [50]	1282	Adolescents aged 12–16, 14-year follow-up	Daily/non- tobacco users	Major depressive disorder: CIDI-SF	11.8%	Physical health, life satisfaction, personal income, years of education	Adjusted: 1.93 (1.01–3.70)
David Brook, 2002, U.S. [52]	736	Adolescents aged 14, A 13-year follow-up	Ever/never tobacco users	Major depressive disorder: Modified version of CIDI	8.3%	Age, sex, parental educational level, family income, prior psychiatric disorders	Adjusted: 1.18 (1.01–1.39)
Longitudinal Studies: Depression (Exposure) → Tobacco Use (Outcome)							
Elizabeth Goodman, 2000, U.S. [42]	6947	Adolescents aged 11–22, 1-year follow-up	Current/non- tobacco users	Depressive symptoms: CES-D, cut-off point of 22 (M) and 24 (F)	6.4%	Age, sex, ethnicity, education	Crude: 2.81 (1.67–4.74)
Kiyuri Naicker, 2012, Canada [51]	681	Adolescents aged 16–17, 10-year follow-up	Daily/non- tobacco users	Major depressive disorder: CIDI-SF, cut-off point of 5	6.9%	Sex and SES	Adjusted: 2.89 (1.53–5.45)
Jie Wu Weiss, 2004, U.S. [37]	1699	Grade 6 students, A 1-year follow-up	Ever/never tobacco users	Depressive symptoms: CES-D, cut-off point of 16	-	change in depression and hostility, sex, ethnicity, SES	Adjusted: 1.76 (1.30–2.38)
Jennifer Mendel, 2012, U.S. [38]	1205	Grade 10–11 students, A 5-year follow-up	Ever/never tobacco users	Depressive symptoms: CES-D, cut-off point of 16	-	Sex, parental marital status, family income, education level, marital status, children, GPA, delinquency, stressful life events, family support, quality of friendship, parental smoking, adolescent alcohol, cannabis use, extent of alcohol problems, peers who drink or use drugs, change in CESD and alcohol use	Adjusted: 0.97 (0.92–1.03)
Jennifer O’Loughlin, 2016, Canada [54]	690	Grade 5 students, 7-year follow-up	Ever/never tobacco users	Depressive symptoms: 6-item question	-	Age, sex, mother’s education	Adjusted: 1.34 (1.16–1.56)
Marcus Munafo, 2007, U.S. [39]	12,149	Grade 7–12 students, 1-year follow-up	Ever/never tobacco users	Depressive symptoms: CES-D, cut-off point of 22 (M) and 24 (F)	-	Age, sex, ethnicity, depressed mood, parental/peer tobacco, alcohol use, delinquency score	Adjusted: 1.13 (1.03–1.25)
William Lechner, 2016, U.S. [43]	2460	Grade 9 students, A 1-year follow-up	Ever/never tobacco users	Depressive symptoms: CES-D, cut-off point of 22 (M) and 24 (F)	-	Age, sex, ethnicity, school, living situation, parental education, use of alcohol and other tobacco products	Adjusted: 1.02 (1.01–1.04)
**First author’s name, year, country**	**Sample size**	Target population	**Substance use measure**	**Mental health disorders assessment**	**% of participants with anxiety**	**Controlled variables**	**OR (95% CI)**
**Association between Anxiety and Alcohol Use**							
**Cross-Sectional Studies**							
Margo Villarosa, 2014, U.S. [29]	532	College students aged 18–22	Alcohol Use Disorder: AUDIT	Social anxiety symptoms: SIAS	-	-	Crude: 1.61 (1.29–2.00)
Meade Eggleston, 2003, U.S. [57]	284	College students aged 17–23	Binge/non-drinkers	Social anxiety symptoms: SIAS	-	-	Crude: 1.55 (1.15–2.10)
Esther Strahan, 2010, U.S. [58]	697	College students aged 17–27	Alcohol Use Disorder: AUDIT	Social anxiety symptoms: SIAS	-	-	Crude: 1.04 (1.01–1.08)
Ping Wu, 2009, U.S. [59]	781	Adolescents aged 13–17	Frequent, heavy/non-drinkers	Any anxiety disorders: DISC	18.4%	Age, ethnicity, public assistance, not living with parents, parental drug/alcohol problems, site	Adjusted: 1.74 (1.07–2.81)
Linda Richter, 2015, U.S. [26]	24,445	Adolescents aged 12–20	Binge/non-drinkers	Any anxiety disorders: Diagnosed anxiety (self-reported)	-	Age, sex, ethnicity	Adjusted: 1.54 (1.12–2.12)
Robert Roberts, 2007, U.S. [35]	4175	Adolescents aged 11–17	Alcohol Use Disorder: AUD	Any anxiety disorders: DISC-IV	6.9%	Mood, conduct oppositional, and ADHD disorders.	Crude: 1.57 (0.72–3.40)
Nancy Low, 2008, U.S. [36]	632	Adolescents aged 13–19	Alcohol Use Disorder: AUD	Any Anxiety Disorders: PRIME-MD	7%	Age, sex, ethnicity, sample site, mood disorders	Adjusted: 3.80 (1.21–11.91)
**Longitudinal Study: Anxiety (Exposure)** → **Alcohol Use (Outcome)**							
Julia Buckner, 2008, U.S. [60]	816	Students aged 15–17, 14-year follow-up	Alcohol Use Disorder: AUD	Any anxiety disorders: K-SADS	-	Sex, conduct, mood, CUDs, T1 AUD excluded	Adjusted: 2.16 (0.82–5.69)
**Association between Anxiety and Cannabis Use**							
**Cross-Sectional Studies**							
Julia Buckner, 2008, U.S. [30]	337	College students aged 18–26	Frequent/non-cannabis users	Social anxiety symptoms: SIAS	18.8%	-	Crude: 1.23 (0.88–1.73)
Robert Roberts, 2007, U.S. [35]	4175	Adolescents aged 11–17	Cannabis Use Disorder: CUD	Any anxiety disorder: DISC-IV	6.9%	Mood, conduct oppositional, and ADHD disorders	Crude: 1.38 (0.70–2.75)
Nancy Low, 2008, U.S. [36]	632	Adolescents aged 13–19	Cannabis Use Disorder: CUD	Any anxiety disorder: PRIME-MD	7%	Age, sex, ethnicity, sample site, mood disorders	Crude: 1.40 (0.40–4.70)
**Longitudinal Study: Anxiety (Exposure)** → **Cannabis Use (Outcome)**							
Julia Buckner, 2008, U.S. [60]	816	Students aged 15–17, 14-year follow-up	Cannabis Use Disorder: CUD	Social anxiety disorder: K-SADS	-	Sex, conduct, mood, AUDs, T1 CUD excluded	Adjusted: 3.28 (1.14–9.40)
**Association between Anxiety and Cannabis Use**							
**Cross-Sectional Studies**							
Gilat Grunau, 2009, Canada [28]	6943	Students aged 13–18	Current/non-cannabis users	Any anxiety disorder: Prescribed anxiety medications	0.6%	Sex, ethnicity, age, parent(s), sibling(s), peer(s) smokes, depression	Adjusted: 1.83 (1.05–3.22)
Ping Wu, 2009, U.S. [59]	781	Adolescents aged 13–17	Daily/non- cannabis users	Any anxiety disorders: DISC	18.4%	Age, ethnicity, public assistance, not living with parents, parental drug/alcohol problems, site.	Adjusted: 3.14 (1.69–5.81)
Amanda Richardson, 2012, U.S. [49]	1884	Adolescents aged 12–15	Ever/never cannabis users	Any anxiety disorder: NIMH-DISC-IV	-	Age, ethnicity, attending school, poverty index ratio, live with smokers, depressive disorder	Adjusted: 4.70 (1.61–13.75)
**Longitudinal Study: Tobacco Use (Exposure)** → **Anxiety (Outcome)**							
Renee Goodwin, 2005, U.S. [34]	940	Students aged 14–18, anxiety: young adults mean age 24.2	Ever/never cannabis users	Any anxiety disorders: K-SADS	-	-	Crude: 1.88 (1.47–2.41)
**Longitudinal Study: Anxiety (Exposure)** → **Tobacco Use (Outcome)**							
Renee Goodwin, 2005, U.S. [34]	940	Students aged 14–18, tobacco use: young adults mean age 24.2	Ever/Never cannabis users	Any anxiety disorders: K-SADS	-	-	Crude: 1.38 (0.83–2.29)

All terms/acronyms used in Table 1 are fully defined in Appendix A.

**Table 2:** Overall pooled estimates for mental health disorders and substance use.

Topics	Study Designs	*n*	OR (95% CI)	*I*^2^% and *p*-Value
Depression and Alcohol use	Overall	9	1.50 (1.24–1.83)	90.54, <0.001
	Cross-Sectional	6	1.57 (1.09–2.26)	92.89, <0.001
	Longitudinal: Alcohol use at baseline	2	1.39 (0.95–2.03)	89.24, <0.001
	Longitudinal: Depression at baseline	1	1.78 (1.10–2.88)	n/a
Depression and Cannabis use	Overall	9	1.29 (1.10–1.51)	74.79, <0.01
	Cross-Sectional	3	1.34 (0.97–1.84)	53.26, 0.12
	Longitudinal: Cannabis use at baseline	5	1.33 (1.19–1.49)	0.00, 0.53
	Longitudinal: Depression at baseline	1	1.03 (1.01–1.05)	n/a
Depression and Tobacco use	Overall	20	1.65 (1.43–1.92)	96.23, <0.01
	Cross-Sectional	10	1.87 (1.55–2.25)	78.60, <0.01
	Longitudinal: Tobacco use at baseline	4	1.87 (1.23–2.85)	92.95, <0.01
	Longitudinal: Depression at baseline	7	1.22 (1.09–1.37)	89.56, <0.001
Anxiety and Alcohol use	Overall	8	1.54 (1.19–2.00)	81.52, <0.001
	Cross-Sectional	7	1.51 (1.16–1.97)	83.28, <0.001
	Longitudinal: Alcohol use at baseline	-	-	-
	Longitudinal: Anxiety at baseline	1	2.16 (0.82–5.69)	n/a
Anxiety and Cannabis use	Overall	4	1.36 (1.02–1.81)	0.39, 0.39
	Cross-Sectional	3	1.27 (0.94–1.70)	0.00, 0.94
	Longitudinal: Cannabis use at baseline	-	-	-
	Longitudinal: Anxiety at baseline	1	3.28 (1.14–9.40)	n/a
Anxiety and Tobacco use	Overall	4	2.21 (1.54–3.17)	46.17, 0.13
	Cross-Sectional	3	2.67 (1.62–4.37)	33.12, 0.22
	Longitudinal: Tobacco use at baseline	1	1.88 (1.47–2.41)	n/a
	Longitudinal: Anxiety at baseline	1	1.38 (0.83–2.29)	n/a

**Table 3:** Subgroup analysis for mental health disorders and substance use.

	Subgroup Analysis		*n*	OR (95% CI)	*I*^2^% and *p*-Value	*p*-Value
**Depression and Alcohol use**	Point Estimate	Crude	3	1.37 (0.87–2.18)	96.08, <0.001	0.56
		Adjusted	6	1.62 (1.19–2.19)	84.60, <0.001	
	Target Population	Adolescents	7	1.69 (1.28–2.25)	90.63, <0.001	0.28
		Young adults	3	1.28 (0.83–1.97)	82.52, <0.001	
	Severity of MHDs ^1^	Symptoms	5	1.37 (1.08–1.75)	94.04, <0.001	0.21
		Disorders	4	1.67 (1.38–2.02)	14.68, 0.32	
	Intensity of substance use	Not binge drinkers	6	1.61 (1.29–2.01)	79.79, <0.001	0.06
		Binge drinkers	6	1.21 (0.99–1.47)	87.67, <0.001	
**Depression and Cannabis use**	Point Estimate	Crude	3	1.27 (0.95–1.69)	80.29, <0.001	0.85
		Adjusted	6	1.31 (1.17–1.46)	0.00, 0.48	
	Target Population	Adolescents	6	1.34 (1.17–1.54)	9.96, 0.35	0.46
		Young adults	4	1.22 (0.99–1.51)	72,62, 0.01	
	Severity of MHDs	Symptoms	5	1.20 (1.01–1.42)	73.16, <0.001	0.16
		Disorders	4	1.41 (1.21–1.65)	0.00, 0.60	
	Intensity of substance use	Cannabis use	7	1.25 (1.06–1.47)	77.10, <0.001	0.23
		CUD ^2^	2	1.63 (1.09–2.44)	0.00, 0.42	
**Depression and Tobacco use**	Point Estimate	Crude	8	1.99 (1.65–2.40)	86.62, <0.001	<0.001
		Adjusted	12	1.30 (1.16–1.45)	87.12, <0.001	
	Target Population	Adolescents	18	1.67 (1.43–1.96)	96.56, <0.001	0.40
		Young adults	3	1.37 (0.89–2.12)	84.18, <0.001	
	Severity of MHDs	Symptoms	14	1.61 (1.36–1.89)	97.18, <0.001	0.48
		Disorders	6	1.91 (1.22–2.97)	78.86, <0.001	
	Intensity of substance use	Ever smokers	7	1.14 (1.04–1.25)	84.89, <0.001	<0.001
		Current smokers	14	1.90 (1.62–2.23)	82.65, <0.001	
**Anxiety and Alcohol use**	Point Estimate	Crude	4	1.37 (1.00–1.88)	86.27, <0.001	0.3
		Adjusted	4	1.70 (1.32–2.18)	0.00, 0.47	
	Target Population	Adolescents	5	1.69 (1.33–2.14)	0.00, 0.64	0.29
		Young adults	3	1.35 (0.96–1.90)	90.40, <0.001	
	Severity of MHDs	Symptoms	3	1.35 (0.96–1.90)	90.40, <0.001	0.29
		Disorders	5	1.69 (1.33–2.14)	0.00, 0.64	
	Intensity of substance use	Alcohol use	6	1.49 (1.26–1.76)	0.00, 0.88	0.59
		AUD ^3^	5	1.71 (1.05–2.79)	83.88, <0.001	
**Anxiety and Cannabis use**	Point Estimate	Crude	2	1.26 (0.93–1.71)	0.00, 0.76	0.19
		Adjusted	2	2.28 (1.00–5.20)	5.37, 0.30	
	Target Population	Adolescents	3	1.71 (1.02–2.89)	0.00, 0.38	0.29
		Young adults	1	1.23 (0.88–1.73)	n/a	
	Severity of MHDs	n/a				
	Intensity of substance use	Cannabis use	1	1.23 (0.88–1.73)	n/a	0.29
		CUD	3	1.71 (1.02–2.89)	0.00, 0.38	
**Anxiety and Tobacco use**	Point Estimate	Crude	1	1.78 (1.42–2.22)	n/a	0.14
		Adjusted	3	2.67 (1.62–4.37)	33.125, 0.22	
	Target Population	n/a				
	Severity of MHDs	n/a				
	Intensity of substance use	Ever	3	1.62 (0.67–3.92)	69.87, 0.04	0.58
		Current	4	2.10 (1.69–2.62)	0.00, 0.52	

^1^ Mental Health Disorders, ^2^ Cannabis Use Disorder, ^3^ Alcohol Use Disorder.

## Appendix A. Definitions of Mental Health Disorders and Substance Use

**Depression**
The center for epidemiologic studies depression scale (CES-D) [27,33,37,38,39,40,41,42,43,44,45]
Beck depression inventory-II (BDI-II) [32]
One item question: Whether respondents felt so sad or hopeless almost daily for two consecutive weeks that usual activities were interrupted [25]
Youth self-report checklist (8 self-reported symptoms of depression within the past 6 months): I feel guilty, I cry a lot, I deliberately try to hurt myself, I think of killing myself, I feel lonely, I am unhappy, I feel that no one loves me, and I feel worthless (YSR) [46]
A five-item question: within the past 12 months how often have you felt too tired to do things? Felt unhappy, sad, or depressed? Felt hopeless about the future? Felt nervous or tense? And worried too much about things? [47]
Modified version of the children depression inventory (CDI) [48]
Depression medication prescribed by a doctor or a nurse in the last 12 months [28]
National institute of mental health diagnostic interview schedule for children version IV (NIMH-DISC, version IV): A structured diagnostic interview administered by lay interviewers to assess diagnostic criteria for mental disorders in children and adolescents, in accordance with the diagnostic and statistical manual of mental disorders, fourth edition (DSM-IV) [49]
CIDI-SF: 12-month prevalence of major depressive disorder (MDD), based on the Composite International Diagnostic Interview–Short Form [50,51]
The university of Michigan composite international diagnostic interview (CIDI): A highly structured research diagnostic instrument developed for use by trained lay interviewers to assess the most common diagnoses among children, adolescents, and young adults as described in the DSM-III-R [52,53]
A 6-item question: During the past 3-month how often you have felt too tired to do things; had trouble going to sleep or staying asleep; felt unhappy, sad or depressed; felt hopeless about the future; felt nervous or tense; worried too much about things [54]
Major depressive episode diagnosis: self-reported depression diagnosed by a medical professional with the past year [26]
The short-form (36) health survey (SF-36) [55]
Mental health component of national health and nutrition examination survey (NHANES), assess diagnostic criteria for mental disorders in children and adolescents, in accordance with the diagnostic and statistical manual of mental disorders, third edition (DSM-III) [31,56]
**Anxiety**
Social interaction anxiety scale (SIAS) [29,30,57,58]
Diagnostic interview schedule for children, version IV (DISC-IV) [26,35,59]
Primary care evaluation of mental disorders (PRIME-MD) [36]
Schedule for affective disorders and schizophrenia for school-age children (K-SADS) [34,60]
Anxiety medication prescribed by a doctor or a nurse in the last 12 months [28]
National Institute of Mental Health Diagnostic Interview for children (NIMHD) [49]
**Alcohol**
Ever drinkers: Drinking at least once within a lifetime [52]
Light drinkers: Consuming less than three drinks per week [31]
Moderate drinkers: Drinking without engaging in heavy episodic or binge drinking in the past two weeks [55]
Moderate drinkers: Drinking no more than 1 to 2 drinks per occasion in the past year, and no intoxication or heavy drinking in the past year [26,44]
Moderate to heavy drinkers: Consuming more than 3 drinks per week [31]
Regular drinkers: Drink at least once a month or more frequently and consume at least three to four drinks when drinking [48]
Current drinkers: Drinking at least one time during the past 30 days [33]
Daily drinking questionnaire (DDQ): The total number of standard alcoholic drinks consumed during the past week by summing the number of drinks reported for each day [29]
Frequent/heavy drinkers: Those who over the past 6 months had a drink at least once a week or had been drunk at least once [59]
Binge drinkers: Men/women as drinking five/four or more drinks per drinking occasion at least once in the past two weeks [26,32,33,44,51,55,57]
Alcohol use disorders identification test (AUDIT) [29,58]
Diagnostic interview schedule for children, version IV, alcohol use disorder (DISC-IV, AUD) [35]
Primary care evaluation for mental disorders, alcohol use disorder (PRIME-MD, AUD) [36]
Longitudinal interval follow-up evaluation and structured clinical interview for DSM-IV, non-patient version, alcohol use disorder (LIFE and SCID-I/NP, AUD) [60]
**Cannabis**
Ever cannabis users: Using cannabis at least once within a lifetime [45,52]
Any use of cannabis or hashish in the past six months [50]
Infrequent cannabis users: Using cannabis at least once per year but not more than once per month [56]
Infrequent cannabis users: Less than weekly cannabis use [30]
Frequent cannabis users: Weekly or more cannabis use [30]
Current cannabis users: Cannabis use at least one time during the past 30 days [27,32,33,40]
The substance abuse module of the composite international diagnostic interview, cannabis use disorder (CIDI-SAM, CUD) [35,36,53,56,60]
**Tobacco**
Ever smokers: Smoking at least once within a lifetime [34,37,38,39,52]
Current smokers: Smoking at least one time during the past 30 days [25,31,32,33,40,41,42,43,46,47]
Regular smokers: Smoking several cigarettes or more per week [48]
Regular smokers: Self-report measure: I’m a regular smoker/I’m a heavy smoker [28]
Daily smokers: Daily consumption of cigarettes or cigars for a continuous 30-day period or longer [28,34,50,51,59]
